# 厄洛替尼治疗晚期非小细胞肺癌分类及回归树分析

**DOI:** 10.3779/j.issn.1009-3419.2011.10.04

**Published:** 2011-10-20

**Authors:** 雨桃 刘, 继红 郭, 燕 王, 娟 杨, 子平 王

**Affiliations:** 1 100021 北京，中国医学科学院北京协和医学院肿瘤医院 Department of Medical Oncology, Cancer Hospital and Institute, Chinese Academy of Medical Sciences and Peking Union Medical College, Beijing 100021, China; 2 610041 成都，四川大学华西公共卫生学院 Department of Epidemiology, West China School of Public Health Sichuan University, Chengdu 610041, China

**Keywords:** 分类及回归树分析, 肺肿瘤, 厄洛替尼, Classification and regression tree analysis, Lung neoplasms, Erlotinib

## Abstract

**背景与目的:**

厄洛替尼是治疗非小细胞肺癌（non-small cell lung cancer, NSCLC）的靶向药物。已有研究表明具有不同临床特征的患者对厄洛替尼的生存获益存在差异，但结论并不一致。本研究将探索厄洛替尼治疗晚期NSCLC生存时间的预测因素以及这些因素的相互影响。

**方法:**

对2006年9月-2009年9月在中国医学科学院肿瘤医院使用厄洛替尼治疗的晚期NSCLC患者的临床及生存资料采用分类及回归树分析（classification and regression tree, CART）。

**结果:**

105例患者的中位无肿瘤进展生存时间（progressive-free survival, PFS）为5.0个月（95%CI: 2.9-7.1）。CART分析将淋巴结分期、厄洛替尼治疗时机及吸烟状况分别作为第一、二、三级划分位点，逐级获得4个终末亚组。生存时间较长的是无淋巴结转移或有淋巴结转移、厄洛替尼治疗≤二线并且吸烟≤35包年的两组患者，中位PFS分别为11.0个月（95%CI: 8.9-13.1）和10.0个月（95%CI: 7.9-12.1），而生存时间较短的是有淋巴结转移且厄洛替尼的治疗>二线或有淋巴结转移、厄洛替尼治疗≤二线并且吸烟>35包年的两组患者，中位PFS分别为2.3个月（95%CI: 1.6-3.0）和1.3个月（95%CI: 0.5-2.1）。

**结论:**

是否存在淋巴结转移、厄洛替尼治疗时机以及既往吸烟状况是影响厄洛替尼治疗PFS的主要因素。CART可以找出既往被我们忽略的亚组患者，有利于为临床实践及今后临床研究找到同质性的患者群体。

## Abstract (en)

**Background and objective:**

Erlotinib is a targeted therapy drug for non-small cell lung cancer (NSCLC). It has been proven that, there was evidence of various survival benefits derived from erlotinib in patients with different clinical features, but the results are conflicting. The aim of this study is to identify novel predictive factors and explore the interactions between clinical variables as well as their impact on the survival of Chinese patients with advanced NSCLC heavily treated with erlotinib.

**Methods:**

The clinical and follow-up data of 105 Chinese NSCLC patients referred to the Cancer Hospital and Institute, Chinese Academy of Medical Sciences from September 2006 to September 2009 were analyzed. Multivariate analysis of progressive-free survival (PFS) was performed using recursive partitioning referred to as the classification and regression tree (CART) analysis.

**Results:**

The median PFS of 105 eligible consecutive Chinese NSCLC patients was 5.0 months (95%CI: 2.9-7.1). CART analysis was performed for the initial, second, and third split in the lymph node involvement, the time of erlotinib administration, and smoking history. Four terminal subgroups were formed. The longer values for the median PFS were 11.0 months (95%CI: 8.9-13.1) for the subgroup with no lymph node metastasis and 10.0 months (95%CI: 7.9-12.1) for the subgroup with lymph node involvement, but not over the second-line erlotinib treatment with a smoking history ≤35 packs per year. The shorter values for the median PFS were 2.3 months (95%CI: 1.6-3.0) for the subgroup with lymph node metastasis and over the second-line erlotinib treatment, and 1.3 months (95%CI: 0.5-2.1) for the subgroup with lymph node metastasis, but not over the second-line erlotinib treatment with a smoking history >35 packs per year.

**Conclusion:**

Lymph node metastasis, the time of erlotinib administration, and smoking history are closely correlated with the survival of advanced NSCLC patients with first- to third-line erlotinib treatment. CART can identify previously unappreciated patient subsets and is advantageous for identifying homogeneous patient populations in clinical practice and future clinical trials.

肺癌是目前全世界死亡率最高的恶性肿瘤，非小细胞肺癌（non-small cell lung cancer, NSCLC）占75%-80%，且诊断时大多已是晚期^[1]^。晚期NSCLC几乎不可治愈，第三代含铂类联合化疗方案虽然仍是一线治疗的标准，但其疗效已经达到平台，中位生存时间仅8个-10个月，1年生存率为30%-40%^[2]^。采用化疗难以进一步提高患者的生存时间。

近年来，表皮生长因子受体（epidermal growth factor receptor, EGFR）小分子酪氨酸激酶抑制剂（tyrosine kinase inhibitor, TKI）吉非替尼、厄洛替尼的出现已经明显地延长了NSCLC患者的生存时间，是治疗NSCLC新的手段。厄洛替尼治疗晚期NSCLC的Ⅲ期临床试验BR.21的数据^[3]^表明，化疗失败的Ⅲb/Ⅳ期NSCLC患者接受厄洛替尼治疗缓解率为8.9%，疾病控制率为45%，并提示厄洛替尼对亚裔、不吸烟的女性腺癌患者疗效较好。尽管目前的研究^[4, 5]^发现，*EGFR*突变对于一线使用厄洛替尼或吉非替尼治疗晚期NSCLC具有决定性的预测价值，然而，尚无足够证据显示使用TKI进行二、三线治疗NSCLC的生存时间与*EGFR*基因突变有明确的相关性。

临床实践中，当患者同时具有良好及不良因素时，多种因素应如何进行分析？是否给予患者靶向治疗？针对这些问题我们对中国医学科学院肿瘤医院采用厄洛替尼治疗晚期NSCLC患者的资料进行了回顾性分析，以期为临床用药提供帮助。

## 1 资料与方法

### 1.1 纳入标准

① 有细胞学或病理学证据的Ⅲb期或Ⅳ期NSCLC（按照IASLC第7版NSCLC TNM临床分期标准）；②既往没有接受过化疗，或曾经化疗过至少一个疗程，且根据RECIST标准进行疗效评估，具有客观疗效评估依据；③一线及化疗后使用厄洛替尼治疗，临床及生存资料完整。

### 1.2 排除标准

① 细胞学或病理学不明确；②临床分期不明或欠缺的病例；③曾有其它肿瘤病史。

### 1.3 临床资料

收集2006年9月-2009年9月中国医学科学院肿瘤医院收治的105例符合上述条件的患者资料。

### 1.4 统计学方法

用SPSS 15.0软件进行统计分析。以开始口服厄洛替尼至有证据出现病情进展或死亡之间的间隔作为无肿瘤进展生存时间（progressive-free survival, PFS），未登记死亡日期的患者，以病历记录的最后日期代替死亡日期，且在生存分析中按照删失数据处理，采用*Kaplan*-*Meier*法描述生存时间的分布情况。本研究以PFS为因变量，性别、年龄、吸烟状况、病理细胞学类型、临床分期、淋巴结转移分期、既往化疗方案是否含有铂类、受累器官数目、合并症数目及TKI治疗时机为自变量进行分类及回归树（classification and regression tree, CART）分析。横向有效分层取值为10，最大深度为3，选择母节点20，子节点8。在进行CART分析时，前述删失数据按照完整数据处理。生存树生长过程中，子结的划分采用杂质缩减最大化的基本思路，对众多自变量进行比较，并筛选出最佳分类变量和最佳分类结果。树的修剪采用成本-复杂度最小原则。

## 2 结果

### 2.1 晚期NSCLC患者临床资料

有105例患者的临床资料符合上述标准，中位年龄为56岁。其中男性69例（65.7%），女性36例（34.3%）； < 70岁90例（85.7%），≥70岁15例（14.3%）。无吸烟史56例（53.3%），>35包年20例（19.0%）。组织学类型包括腺癌74例（70.5%），非腺癌31例（29.5%），其中鳞癌19例（18.1%）、肺泡癌5例（4.7%）、其它7例（6.7%）；Ⅲb期、Ⅳ期患者分别为9例（8.6%）和96例（91.4%）；无淋巴结转移18例（17.2%），有淋巴结转移87例（82.8%），其中N1、N2、N3分期分别有2例（2.3%）、55例（63.2%）及30例（34.5%）；0、1个、2个、3个、4个和5个脏器转移的分别为7例（6.7%）、23例（21.9%）、25例（23.8%）、32例（30.5%）、12例（11.4%）和6例（5.7%）；曾经使用含铂类化疗方案的91例（86.7%），非铂类方案1例（0.9%）；厄洛替尼一线治疗13例（12.4%），二线治疗40例（38.1%），≥三线52例（49.5%）。

### 2.2 生存情况

105例晚期NSCLC患者的中位PFS为5.0个月（95%CI: 2.9-7.1），*Kaplan*-*Meier*生存曲线见图 1。

**1:**
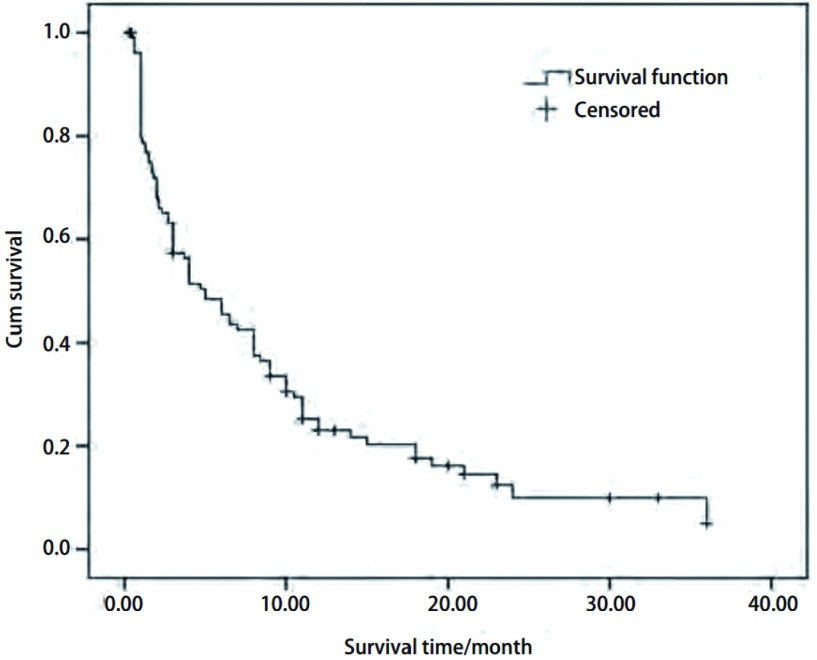
105例晚期非小细胞肺癌患者无肿瘤进展生存时间生存曲线。中位无肿瘤进展生存时间为5.0个月（95% CI: 2.9-7.1） *Kaplan*-*Meier* survival curve of 105 cases of advanced non-small cell lung cancer (NSCLC). The median progressive-free survival (PFS) was 5.0 months (95%CI: 2.9-7.1)

### 2.3 CART分析结果

CART分析将是否有淋巴结转移作为第1个划分位点，厄洛替尼治疗时机作为第2个划分位点（≤二线及>二线），吸烟状况作为第3个划分位点。逐级划分后获得了4个末端结，分别是第1、4、5、6亚组（图 2）。4个终末亚组的生存曲线见图 3。

**2:**
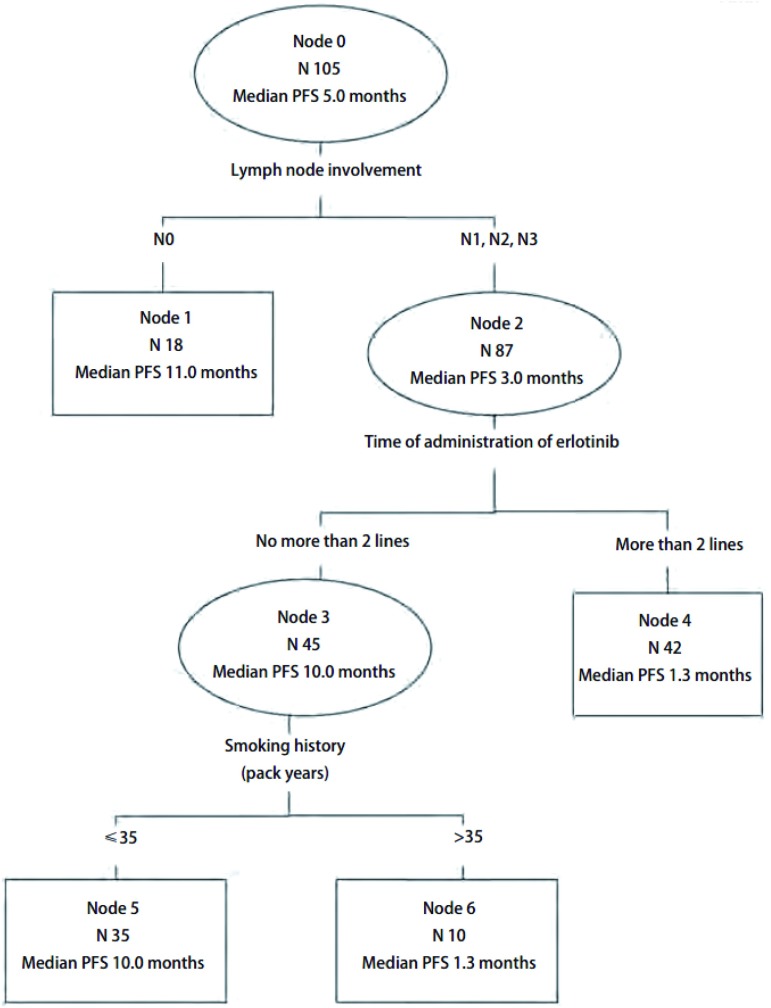
分类及回归树图形，首个划分位点为淋巴结分期，次级划分位点为厄洛替尼治疗线数，三级划分位点为吸烟状况 CART generated with the initial split on the outcome of the stage of lymph node, and then, on the time of administration of erlotinib, and on smoking status

**3:**
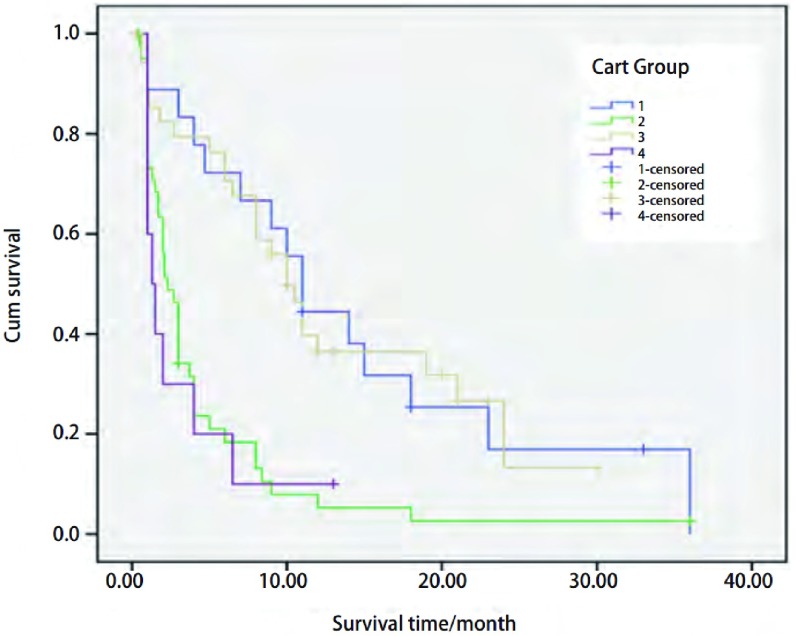
CART分析后4个终末亚组*Kaplan*-*Meier* PFS生存曲线（其中1-4依次代表前述第1、第4、第5、第6亚组），第1、第4、第5、第6组的中位PFS分别为11.0个月、2.3个月、10.0个月、1.3个月 *Kaplan*-*Meier* survival curves of the 4 terminal subgroups generated from the CART analysis (Node1、4、5、6 indicated by 1-4). Median PFS of the first、forth、fifth、sixth subgroup was 11.00, 2.30, 10.0, 1.30 months respectively

生存时间较长的亚组是第1、第5亚组，为无淋巴结转移或有淋巴结转移、厄洛替尼的治疗≤二线并且吸烟≤35包年的两组患者，中位PFS分别为11.0个月（95%CI: 8.9-13.1）和10.0个月（95%CI: 7.9-12.1），而生存时间较短的第4、6亚组，为有淋巴结转移且厄洛替尼的治疗>二线，或有淋巴结转移、厄洛替尼的治疗≤二线并且吸烟>35包年的两组患者，中位PFS分别为2.3个月（95%CI: 1.6-3.0）和1.3个月（95%CI: 0.5-2.1）。患者淋巴结转移情况、吸烟状态和厄洛替尼使用时机与PFS关系密切，无淋巴结转移或厄洛替尼的治疗≤二线并且吸烟≤35包年的患者无进展生存时间明显长于厄洛替尼的治疗>二线或吸烟>35包年的患者。

## 3 讨论

厄洛替尼是口服EGFR-TKI，既往研究^[3]^发现其可以明显延长化疗失败的晚期NSCLC患者的生存，亚组分析发现一些具有某些临床特征的患者拥有更多的生存机会。BR.21研究^[3]^显示，厄洛替尼组患者中位生存期为6.7个月，相比安慰剂组的中位生存期（4.7个月）改善了42.5%，两组相比有统计学差异，该研究同时也说明厄洛替尼对亚裔不吸烟的女性腺癌患者的疗效较好。

Lynch等^[6]^首先揭示了*EGFR*基因突变与TKI疗效之间的关系，随后的一些研究^[7]^也显示*EGFR*基因突变与TKI疗效较好的临床特征相关，例如：吸烟与否和*EGFR*基因突变呈明显的相关性，提示可以将这些临床因素看成是*EGFR*基因突变的替代指标^[8]^。IPASS研究^[4]^显示吉非替尼一线治疗具有*EGFR*基因敏感突变的晚期NSCLC较紫杉醇/卡铂方案疗效更为突出，PFS具有明显优势。Rosell等^[5]^报告厄洛替尼一线或二线治疗*EGFR*基因敏感突变的晚期NSCLC治疗总有效率为73.1%，所有患者总生存期（overall survival, OS）为24个月。然而，尽管TKI二线及三线治疗对化疗失败后的NSCLC是一个合理的选择，但到目前为止尚无足够证据显示*EGFR*的突变状况与二线及三线治疗疗效之间存在必然联系。一线应用TKI的研究结果不能照搬到二线及二线以上的治疗之中，二线及以上治疗优势人群的概念对药物的选择仍起着深刻的影响。一些研究^[9]^显示非优势人群中也存在基因突变，例如：曾经或正在吸烟患者的*EGFR*基因突变率为23%，因此，单独就某项非优势或优势指标就拒绝或接受使用厄洛替尼就有可能出现忽略或夸大正确使用TKI的范围。其次，具备所有优势因素的患者是很少的，更多患者同时具备优势和劣势因素，因此很难将几个因素结合起来综合预测患者的预后。

有研究^[10]^发现结合多个基因较单基因更能准确预测TKI的疗效。临床也应该开展多因素的研究以利于临床需要。目前对恶性肿瘤等多因素疾病的研究多采用如多元线性回归分析、*Logistic*回归、*Cox*回归等模型进行危险因素的筛选。这些方法多要求样本来自同一总体，具有同质性，但许多临床资料很难满足这一条件，因为同种疾病的一组患者其内部同质性通常较差。CART分析不受上述条件的约束，是一种既包含了多种多因素统计分析方法的优点又能克服其缺陷的新的统计分析方法，可以将不同特征的个体分配到树的各个局部去处理，使每个局部样本的同质性得到改善。CART的树型结构与临床思维十分接近，体现了生存特征，通过分级树的级数来阐明各研究变量的重要性。

本研究的105例患者一线及二、三线使用厄洛替尼治疗的中位PFS为5个月，与以往的研究^[3]^结果相似。是否存在淋巴结转移、厄洛替尼治疗时机以及既往吸烟状况是影响厄洛替尼治疗PFS的主要因素。淋巴结转移对NSCLC预后有明显影响，临床资料^[11]^表明，术后病理淋巴结（N）分期pN0、pN1、pN2和pN3的中位生存期分别为77个月、34个月、21个月和12个月，5年生存率为56%、38%、22%和6%，不同组间比较差异均具有统计学意义。本研究入选的虽然均为晚期NSCLC患者，但淋巴结转移作为预后影响因素在CART分析中率先筛选出来，其对预后比较明显的影响得到了进一步印证。在有淋巴结转移的患者中，在≥三线治疗中再选择厄洛替尼治疗预示着预后欠佳，这可能也提示，对于基因状态不明的患者EGFR-TKI药物越早应用越能够改善患者的生存。但2010年ASCO会议上Gridelli^[12]^报道的TORCH研究，采用交叉试验设计，在未经选择的患者中比较一线吉西他滨+顺铂/二线厄洛替尼方案（标准治疗组）与一线厄洛替尼/二线吉西他滨+顺铂方案（试验组）的疗效，结果显示标准治疗组的PFS优于实验组（5.7个月*vs* 2.2个月），OS（10.9个月*vs* 7.7个月，HR=1.40）结果也支持先化疗后用厄洛替尼治疗。TORCH研究是在白种人、非选择人群中开展的研究，这部分人群80%-90%是*EGFR*野生型患者，其靶向治疗有效率均 < 10%，而亚裔人群*EGFR*突变率普遍高于白种人，基因突变率较高。因此TORCH研究结果与本研究所提示一线或二线使用厄洛替尼治疗改善生存的结果并不矛盾。有报道^[9]^既往吸烟超过15包年患者的*EGFR*突变率明显下降至9%以下，本研究CART分析中也出现了吸烟包年这一对预后有明显影响的因素，不过是既往吸烟超过35包年对PFS存在影响，考虑到本研究属回顾性研究，样本量相对偏少，此外患者其它因素如性别等可能也对结果产生了影响。

在本研究中腺癌、女性因素并没有在回归树分析中出现，其原因为105例患者绝大多数病理类型就是腺癌，进行多因素分析后，或许这些优势人群多存在于生存时间较长的亚组之中。当患者的样本量进一步扩大后可能还会出现更多的影响因素。使用TKI后生存获益最多人群的组织标本资料将会为基础研究提供珍贵的信息，对这一领域深入的了解，将会对临床治疗提供更加有力的依据。目前，*EGFR*基因突变对二线及以后使用TKI治疗NSCLC生存结果的影响尚缺乏足够的依据，临床工作中能根据临床特征间接预测可能敏感的患者就显得非常重要。

在本研究中生存时间较长的无淋巴结转移和有淋巴结转移、厄洛替尼的治疗≤二线并且吸烟≤35包年的两组患者生存曲线接近，生存时间较短的有淋巴结转移且厄洛替尼的治疗>二线或有淋巴结转移、厄洛替尼的治疗≤二线并且吸烟>35包年的两组患者生存曲线也比较接近，在临床上，这是不同人群，提示生存特征有可能相似，但可能其内部还有某些根本的不同才被模型分到不同的亚组，也许是因为样本量有限尚未表现出来所致。

综上所述，本研究对105例NSCLC患者采用CART分析方法进行临床信息的分析，发现是否存在淋巴结转移、厄洛替尼治疗时机以及吸烟状况是影响厄洛替尼治疗PFS的主要因素，此结果还需要进一步扩大样本量来进行验证。
